# Trajectory of vitamin D, micronutrient status and childhood growth in exclusively breastfed children

**DOI:** 10.1038/s41598-019-55341-1

**Published:** 2019-12-13

**Authors:** Sui-Ling Liao, Tsung-Chieh Yao, Man-Chin Hua, Ming-Han Tsai, Shih-Yun Hsu, Li-Chen Chen, Kuo-Wei Yeh, Chih-Yung Chiu, Shen-Hao Lai, Jing-Long Huang

**Affiliations:** 1 Community Medicine Research Center, Chang Gung Memorial Hospital at Keelung, Keelung, Taiwan; 2 Department of Pediatrics, Chang Gung Memorial Hospital at Keelung, Keelung, Taiwan; 3 Division of Allergy, Asthma, and Rheumatology, Department of Pediatrics, Chang Gung Memorial Hospital, Taoyuan, Taiwan; 4 Division of Pulmonology, Department of Pediatric, Chang Gung Memorial Hospital, Taoyuan, Taiwan; 5 Chang Gung University, College of Medicine, Taoyuan, Taiwan

**Keywords:** Nutrition disorders, Health care

## Abstract

This study aimed to compare the trajectory of serum 25(OH)D, micronutrient levels, and anthropometric measurements between exclusively breastfed and mixed-fed children. This is a prospective cohort study. Anthropometric measurements of the children were obtained during scheduled clinical visits. Tests for 25(OHD), ferritin, zinc and complete blood count were performed yearly until 3 years of age. Clinical records and questionnaires on dietary habits were obtained. The results showed that despite official recommendations on vitamin D/iron supplements for breastfed children, less than 10% of our exclusively breastfed children received regular supplements. Thus, after 1 year, the odds for having iron deficiency anemia and vitamin D insufficiency were 9 [95% CI, 4–19] and 6 [95% CI, 2–16], respectively. Longitudinal follow-up showed the prevalence of iron deficiency to decrease from 34% at 1 year to 2% at age 3 years. However, the prevalence of vitamin D insufficiency remained persistently high throughout the first three years of life (60% at 1 to 44% at 3 years). Very few children had zinc deficiency. Anthropometric measurements showed exclusively breastfed children to have lower mean *z*-scores for body weight and height when compared to mixed-fed children after 12 months. In conclusion, children who were exclusively breastfed for longer than 4 months without proper supplement were more likely to have transient iron deficiency anemia and persistent vitamin D insufficiency. Their growth became relatively slower after infancy. Whether this was associated with underlying inadequate serum vitamin D and iron level remains an important issue to be explored.

## Introduction

Benefits of breastfeeding are unquestionable, and breast milk contains all the necessary nutrients for proper infant growth. However, despite innumerable nutritional advantages, emerging reports have shown that prolonged or exclusive breastfeeding was associated with certain nutrient deficiency such as iron^1,2^, zinc^3,4^, and vitamin D^5–8^. Thus, various organizations such as the Center for Disease Control and Prevention (CDC), World Health Organization (WHO), American Academy of Pediatrics, Canadian Paediatric Society, and the Taiwanese Pediatric Association recommend breastfed infants be supplemented with 400 IU per day of vitamin D beginning in the first few days of life and iron drops or iron-enriched foods at 6 months^9,10^ However, how well the caregivers follow such recommendation is unknown, and nutritional status in these exclusively breastfed children remained undetermined. Accordingly, the primary aim of this study was to investigate the trajectory of nutritional status such as vitamin D and other important micronutrients in infants who were exclusively breastfed for at least 4 months.

Early childhood is a critical period where nutritional status and physical growth are important foundations for future well-beings. Nutrients such as vitamin D, iron, and zinc are essential elements required to achieve proper health and growth in children. Whether deficiency in these important nutrients have any effect on body growth deserves further investigation. In addition, reports have shown that infants who were exclusively breastfed for longer than 4–6 months showed a persistent deceleration in both weight and length gain when compared to formula-fed children. Hence, the World Health Organization (WHO) had subsequently integrated a set of normative curves from the Multicenter Growth Reference Study (MGRS), and developed a new growth curve standard that established the breastfed infants as the normative model for growth and development^11,12^. Thus, by using the new WHO growth standards, the second aim of this study was to determine whether extended exclusive breastfeeding was still associated with lower growth parameters during the first 3 years of life. Results from this cohort study might provide a better understanding of current nutritional status in exclusively breastfed infants and their growth patterns during early childhood.

## Results

### Subject characteristics

**Figure 1:**
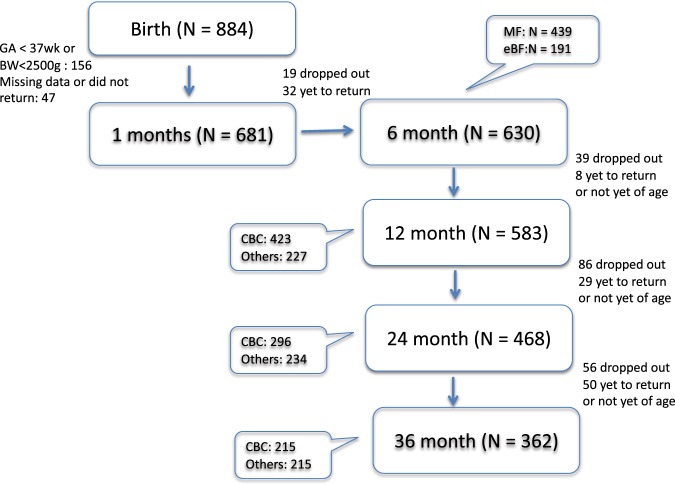
Flow chart of the birth cohort study: demonstrating the number of participants at each age point with complete questionnaire information and anthropometric measurements. Number of blood analyses is as shown in the figure: CBC: complete blood count. Others: includes tests for serum ferritin, zinc, and 25-hydroxyvitamin D [25(OH)D] level.

By 6 months of age, 630 infants were enrolled in this analysis. We separated the study subjects into 2 groups according to their feeding habits. The exclusive breastfed group (eBF group) had 191 children with mean breastfeeding duration of 12 ± 6 months. The mixed-fed group (MF group) comprised of 439 children, and the mean duration of breastmilk given was for approximately 2 months. All children were followed as scheduled until 3 years of age. Complete questionnaires and anthropometric measurements were available for all study subjects at each age point as shown in Fig. 1. While due to difficulties or refusals in drawing blood samples, not all children had blood test results. Detailed number of participants and test samples at each age point was listed in Fig. 1. By 3 years of age, 268 children were lost to follow-up or lacked complete data. As shown in Supplement 1, except for slight discrepancy in gestation age, no differences were found between the participants who continued in the study and those lost to follow-up. Characteristics of the participants were summarized in Table 1. Except for breastfeeding duration and maternal academic degrees, there was little difference between children of the eBF or MF group. Mothers with higher education tended to exclusively breastfeed their children for a longer period of time. Very few infants received the recommended iron/vitamin D supplements during periods of exclusive breastfeeding (only 9.7% of the children received vitamin D_3_ and 7% had iron drops).

**Table 1:** Demographic characteristic of infants of the different feeding group.

Characteristics	MF n (%)	eBF n (%)	p
Sex (male)	242 (55)	95 (50)	0.22
Gestational Age (wk)	38.6 ± 1	38.7 ± 1	0.06
Birth body weight (g)	3160 ± 400	3210 ± 360	0.15
Birth body height (cm)	50.5 ± 2	50.8 ± 2	0.13
HC at birth (cm)	33.8 ± 1.3	34.0 ± 1.2	0.10
Mode of delivery (NSD)	271 (62)	131 (70)	0.10
Mean BF duration (mo)	2.1 ± 4	12.0 ± 6	<0.001
Time of solid food (mo)	5.1 ± 1	5.2 ± 1	0.20
Maternal Allergy	140 (32)	66 (35)	0.51
Maternal body height (cm)	160 ± 6	159 ± 5	0.27
Maternal body weight (kg)^*^	59.5 ± 10	59.8 ± 12	0.91
Matermal BMI	23.2 ± 3.8	23.3 ± 3.5	0.94
Maternal Education			<0.001
Secondary or high school	143 (33)	31 (16)	
College or above	296 (67)	160 (84)	
Infant vitamin D supplement	NA^**^	18 (9.7)^***^	NA
Infant iron supplement	NA	13 (7)	NA

eBF: exclusively breastfed.

MF: mixed-fed.

BF: breastfeeding.

HC: Head circumference.

NSD: natural spontaneous delivery.

mo: months wk: weeks.

NA: non-available.

Total number of children in the MF group: 439 (including any children who were formula-fed or formula were given in conjunction to breast milk within the first 4 months).

Total number of children in the eBF group: 191 (children who were exclusively breastfed for longer than 4 months).

* Maternal body weight was the average weight before pregnancy.

**Since most formula contain vitamin D and iron supplement, data were not collected for infants of the MF group.

***Percentage was calculated based on the number of infants of the eBF group at 1 year of age (N = 185) who received regular iron/vitamin D supplements during periods of breastfeeding.

### Comparing complete blood count, serum 25(OH)D, and micronutrient concentration between children of the eBF and MF group at 1 year of age

Complete blood count and serum concentration of 25(OH)D, ferritin, and zinc were examined and compared between infants of the eBF and MF group at 1 year of age. Since low iron status is often associated with anemia, hemoglobin and hematocrit were also analyzed. White blood cell count was also compared to rule out any possible associated infection that might affect serum ferritin level. The result showed eBF children to have significantly lower hemoglobin/hematocrit, serum ferritin and 25 (OH)D concentration at age 1 year when compared to MF children. No difference was noted in white blood cell count and serum zinc concentration (Table 2). To further examine the status of nutrient deficiency in children of the eBF group, binary regression analysis was performed by using cut-off levels at 11 g/dl for hemoglobin and 10 ng/mL for serum ferritin to determine the odds of having iron-deficiency anemia. Vitamin D insufficiency was defined as having serum 25(OH)D level < 30 ng/mL. The result showed that exclusive breastfeeding without adequate supplementation was associated with increased risk of having iron deficiency anemia and vitamin D insufficiency at 1 year of age. Adjusted OR for Hb was 9 [95% CI, 4–19]), for serum ferritin was 15 [95% CI, 1–180], and for 25(OH)D, it was 6 [95% CI, 2–16]) (Table 3).

**Table 2:** Comparing blood count and serum micronutrient concentration between infants of the different feeding groups at 1 year of age.

Blood indices	MF	eBF	P
WBC (1000/uL)	9.5 ± 2.6	9.0 ± 2.3	0.12
Hemoglobin (g/dL)	12 ± 2.2	11 ± 3.8	<0.001
Hematocrit (%)	36 ± 2.3	34 ± 3.8	<0.001
Ferritin (ng/mL)	46 ± 38	18 ± 11	<0.001
Zinc (ng/mL)	80 ± 13	80 ± 10	0.78
25(OH)D (ng/mL)	40 ± 11	29 ± 12	<0.001

WBC: white blood count.

Analysis by student *t* test.

**Table 3:** Effect of exclusive breastfeeding for longer than 4 months on iron deficiency anemia and vitamin D insufficiency at 1 year of age.

	Crude OR (95% CI)	p	Adjusted OR (95% CI)	p
Hb ≤ 11 g/dl	7 (4–13)	<0.001	9 (4–19)	<0.001
Fe ≤ 10 ng/ml	9 (3–32)	<0.001	15 (1–180)	0.03
25(OH)D ≤ 30 ng/ml	7 (3–13)	<0.001	6 (2–16)	<0.001

Hb: hemoglobin; Fe: ferritin.

Binary logistic regression analysis.

OR: odds ratio: adjusted for birth body weight, gestational age, mode of delivery, gender, time of solid food introduction, and maternal education.

95% CI: 95% confidence interval.

### Comparing the trajectory of hemoglobin, vitamin D, and micronutrient status between children of the eBF and MF group during early childhood

**Figure 2:**
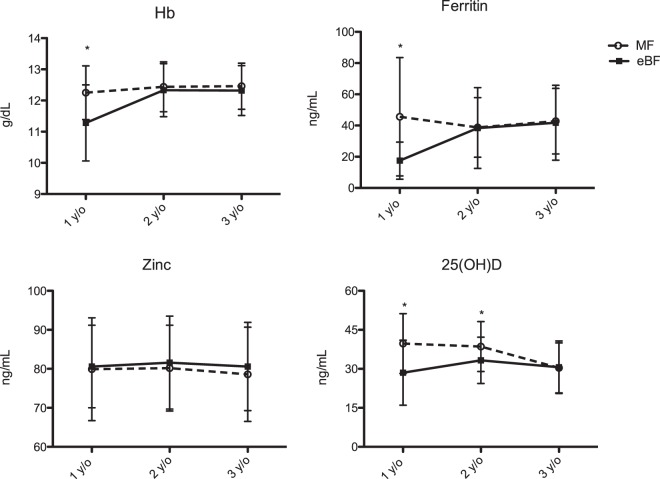
Comparing the trajectory of hemoglobin (Hb), serum ferritin, zinc, and 25(OH)D level between children of the eBF (solid line) and MF group (dotted line) during the first three years of life. Values represent means ± SD; *p < 0.05.

Longitudinal follow up of serum hemoglobin and micronutrient levels were followed yearly until 3 years and compared between the two groups (Fig. 2). Results showed the eBF children to have significantly lower serum ferritin and hemoglobin at 1 year of age, but gradually increased to a level comparable to that of the MF children after 2 years. The percentage of having iron deficiency in children of the eBF group was 34%, 4%, and 2% at ages 1, 2, and 3 years, respectively; as oppose to 5%, 2%, and 2% in children of the MF group. Serum 25(OH)D concentration was persistently lower in eBF children. The prevalence of having vitamin D insufficiency was 60% at age 1, 40% at ages 2, and 44% by ages 3 years. Although serum 25(OH)D concentration was significantly higher in children of the MF group during the first 2 years, their serum level had dropped to a level similar to that of the eBF group by age 3 years. Very few children in our study had zinc deficiency. Both groups had comparable serum zinc levels throughout the first 3 years of life.

### Comparing anthropometric measurements between eBF and MF children

Nutrients such as vitamin D, iron, and zinc are essential elements required to achieve proper growth in children. Because we had documented lower serum ferritin and 25(OH)D concentration in our breastfed children, we had then proceeded to analyze the trajectory of their growth pattern. We assessed the growth of our children based on the WHO’s new international growth standard that established breastfed infant as the normative model for growth and development. As a developed country, we had very few children with stunted growth as most children had growth parameters within the WHO growth standards (data not shown). Comparison of anthropometric measurements were performed at ages 6, 12, 24, and 36 months between children of the different feeding groups. Results showed comparable growth parameters at 6 months between the eBF and the MF infants. However, after 12 months, the mean *z*-scores for body weight and length became significantly lower in children of the eBF group when compared to children of the MF group. These eBF children remained persistently shorter and lighter-weighted for the first three years of life. No differences were found in head circumference and BMI measurements (Table 4).

**Table 4:** Comparing *z*-scores for weight, length, head circumference, and BMI between children of different feeding habit.

	MF group	eBF group	p
***6 months of age***			
Weight-for age	0.21 ± 1.01	0.16 ± 0.99	0.74
Length-for age	0.15 ± 1.11	−0.41 ± 1.05	0.14
Weight-for length	0.32 ± 1.07	0.35 ± 1.04	0.59
HC-for age	0.18 ± 1.01	0.19 ± 1.00	0.98
BMI-for age	0.21 ± 1.05	0.25 ± 1.05	0.66
***1 year of age***			
Weight-for age	0.14 ± 0.94	−0.08 ± 0.91	0.01
Length-for age	0.03 ± 1.11	−0.27 ± 1.02	0.003
Weight-for length	0.19 ± 0.96	0.06 ± 0.92	0.13
HC-for age	0.29 ± 0.93	0.14 ± 0.96	0.09
BMI-for age	0.18 ± 0.96	0.11 ± 0.91	0.39
***2 years of age***			
Weight-for age	0.18 ± 1.00	−0.09 ± 0.99	0.02
Length-for age	0.02 ± 1.02	−0.38 ± 1.06	0.001
Weight-for length	0.19 ± 1.07	0.12 ± 1.02	0.53
HC-for age	0.31 ± 0.92	0.16 ± 0.93	0.18
BMI-for age	0.21 ± 1.09	0.19 ± 1.05	0.83
***3 years of age***			
Weight-for age	0.09 ± 0.73	−0.11 ± 0.62	0.003
Length-for age	−0.05 ± 0.73	−0.26 ± 0.70	0.003
Weight-for length	0.36 ± 1.21	0.09 ± 0.95	0.05
HC-for age	0.26 ± 0.83	0.09 ± 0.15	0.72
BMI-for age	0.17 ± 0.89	0.06 ± 0.73	0.16

*z*-scores were calculated by using the World Health Organization growth curve standards.

HC: head circumference.

BMI: body mass index.

Values are means ± standard deviation (SD).

*p* values were analyzed by student *t* tests.

## Discussion

Results from this study showed that despite official recommendations on vitamin D and/or iron supplement for exclusively breastfed infants, adherence to guideline recommendation was low. Thus, children who were exclusively breastfed for longer than 4 months had higher prevalence of having iron deficiency anemia at 1 year and vitamin D insufficiency throughout the first 3 years of life. Trajectory of growth pattern showed eBF children to begin having lower mean *z* scores for body weight and height after 1 year of age and remained persistently smaller at 3 years when compared to children of the MF group. However, albeit being smaller, eBF children had growth parameters well within the new WHO standards. To our knowledge, this is the first cohort study to simultaneously determine the trajectory of both nutrient status and growth pattern in exclusively breastfed children residing in an economically- advantaged country.

From the current study, we had realized that only around 10% of our infants received the advocated vitamin D or/and iron supplements during the period of exclusive breastfeeding. The reasons were that numerous caregivers had no knowledge of such recommendations; others believed that their breastmilk contained sufficient nutrients for their infants and consider supplements to be artificial or unnecessary; and finally, many had ignored the risks of vitamin D and iron deficiency, as children with iron deficiency anemia or vitamin D insufficiency are often asymptomatic, and clinical cases of rickets are uncommon. With our results indicating a high prevalence of vitamin D and iron deficiency, we emphasize the importance of constant parental education about complementing iron during the first year and vitamin D supplements for at least 3 years in children with extended breastfeeding. In addition, maternal vitamin D supplementation (6000–6400 IU/day) has been reported to safely supply breast milk with adequate vitamin D to maintain proper infant serum 25(OH)D, thus, might serve as an alternative strategy to direct infant supplementation^13,14^

Nevertheless, it remained questionable whether nutrient deficient status in these otherwise healthy breastfed infants has any clinical implication or adverse health outcome. Yet, since vitamin D and iron have been identified to have critical effects on body growth, we had subsequently investigated the trajectory of growth pattern in our subjects. Our findings regarding body weight was consistent with several studies that also demonstrated lighter weight in breastfed infants when compared to formula-fed infants^15–17^. Rapid and excess weight gain during the first year of life was reported to be a predictor of later obesity. Thus, our result had shown that children who were exclusively breastfed would most likely be protected from future obesity^18–20^. In contrast to body weight that showed universally smaller weight in breastfed infants, reports on body height were more conflicting. Some reports showed no difference in mean z-scores for height between breastfed and formula-fed infants^17,21^. However, similar to our observation, several studies had shown exclusive breastfeeding to be associated with shorter stature^15,16,22,23^. Our study too had observed lower z scores for body height in eBF children. Whether the relatively decelerated growth in these eBF children was associated with underlying inadequate serum iron and vitamin D level deserves further investigation.

Yet, to explain the reason for decelerated growth, one had to consider the effect of complementary food consumption. As seen in Supplement 2, the time and variety of solid food introduction were similar between both groups. Although children of the eBF group had a slightly higher rate of inadequate solid food consumption, the difference was not significant. For children of the MF group, since most formulas were fortified with vitamin D and iron (mostly 400IU of vitamin D and 4–12 mg of iron per liter), those who did not receive proper solid food might not immediately suffer from consequences of micronutrient deficiency, thus able to transiently maintain proper body growth. However, for children with extended breastfeeding, inadequate amount of solid food consumption might aggravate nutrient (vitamin D and iron) deficiency, thus, if supplement was not given in addition to breastmilk, the compounded effect might have resulted in persistent deficient status and subsequent decelerated growth.

We had assumed that insufficient serum nutrient level might be related to the decelerated growth in our breastfed children based on the fact that iron affects body growth through its role in hemoglobin concentration, tissue oxygenation, and induction of insulin-like growth hormone binding protein −1 (IGFBP-1) that stimulates cell proliferation^24^; hence, inappropriate serum iron level might deter proper growth. In addition, reports have shown low serum 25(OH)D to be associated with slower linear growth in children; thus, supplementation with vitamin D had resulted in accelerated body height, possibly through its effect on serum insulin-like growth factor - 1 (IGF-1) concentrations and bone turnover rate during growth^25–28^, although a recent randomized placebo-controlled trial study had concluded that neither prenatal nor postpartum vitamin D supplementation was effective in improving infants’ length-for-age z scores at 1 year^29^. Nevertheless, their growth outcome assessment was performed only up to the age of 1 year. Our study had shown comparable height measurements between 6 –months-old infants from either group. Growth difference only became more evident after 1 year. Therefore, the role of vitamin D on the trajectory of childhood growth beyond infancy remains to be further explored.

Despite our strength as a prospective cohort study with valuable longitudinal data, our study had several limitations. First, we did not have data on detailed contents and precise quantities of solid food intake; thus, it is possible that body growth was related to dietary habit or total energy intake rather than nutrient deficiency. Next, results would be more ideal if comparisons were made simply between exclusive breastfed or formula-fed infants. By including partially breastfed infants, we might had inadvertently included a few infants who had been exclusively breastfed for nearly 4 months into the MF group. However, nowadays, many infants are partially breastfed, to exclude these infants from our analysis would have left us with very few subjects and might not reflect the real scenario of current feeding practices. Finally, we have not evaluated the consequences of iron deficiency anemia and vitamin D insufficiency on our breastfed children in regard to cognitive performances and other health issues, because whether any clinical implication of having lower than the reference interval data have on future well-being of these eBF children warrants further exploration.

## Conclusion

Our results showed that children who were exclusively breastfed for longer than 4 months without adequate vitamin D and/or iron supplement had higher prevalence of iron deficiency anemia at 1 year and persistent vitamin D insufficiency throughout the first 3 years of life when compared to children who were mix-fed. Although these exclusively breastfed children were smaller than mixed- or formula-fed children, their growth parameters were within the new WHO growth standards. With our results indicating a high prevalence of deficient status due to low supplemental rate, we highlighted the importance of constant parental education about complementing iron during the first year and vitamin D supplements for at least 3 years in children with extended breastfeeding. However, whether regular supplementation would have any effect on the trajectory of childhood growth would require further investigation.

## Methods

### Study population

Data for this analysis came from an ongoing prospective birth cohort study called the PATCH (The Prediction of Allergy in Taiwanese Children). The 3^rd^ phase of the PATCH study was launched in March 2015 in Keelung city (25° N latitude). Data for current analysis were collected by the end of September 2018. The study was approved by the Chang Gung Ethics Committee and all methods performed in the research were in accordance with the relevant guidelines and regulations. Written informed consent was obtained from the parents/legal guardians of the neonates. Neonates with low birth body weight (<2500 g), under the gestational age of 37 weeks, had major congenital anomaly or chronic illnesses likely to affect growth were excluded from this analysis. This study comprised of the first 681 eligible healthy full-term infants who returned to the health clinic at 1 month of age and were confident about their feeding preferences. Initial questionnaire survey included perinatal information, obstetric history, parental allergy, socio-economic status, smoking history, and etc. Standardized questionnaires on feeding practices, complementary food introduction, and environmental factors were answered at 1, 4, 6,12, 18, 24, and 36 months.

### Grouping

Detailed information regarding feeding habits and breastfeeding duration was recorded at each visit. Infants who were fed with breast milk only for longer than 4 months were enrolled in the exclusive-breastfed group (eBF group). Those who were formula-fed or infant formula were given in conjunction to breastmilk during the first 4 months of life were enrolled in the mixed-fed group (MF group). In this study, exclusive breastfeeding was defined at the cut-off point of 4 months, because it is in agreement with the international recommendations on breastfeeding, and many infants have started complementary food by 4–6 months of age^12,30,31^.

### Complete blood count, serum 25(OH)D, ferritin, and zinc measurement

Peripheral blood was collected at the ages of 12, 24 and 36 months. Samples were sent to the central clinical laboratory to test for complete blood count (CBC), serum 25(OH)D, ferritin, and zinc level. Serum 25(OH)D level was measured by Elecsys Vitamin D total assay (Roche Diagnostics, Mannheim, Germany). This method is a new automated electrochemiluminescense-based assay that measures both the 25(OH)D_2_ and 25(OH)D_3_ as total 25(OH)D level. Serum ferritin level was measured by ADVIA Centaur Ferritin assay, a two-sited sandwich immunoassay using direct chemiluminometric technology. Zinc analysis was performed by atomic absorption (AA) spectroscopy by PerkinElmer PinAAcle 900 T (PerkinElmer SCIEX USA). To avoid the effect of infection on measured values, drawing of blood samples were delayed if children had any signs of infection (including fever, rhinorrhea, cough, diarrhea, or any other discomforts suspicious of infection) within 3 weeks of blood testing.

### Anthropometric measurement

Anthropometric measurements were obtained during scheduled clinical visits by nursing staff that were trained in appropriate techniques for anthropometry measurement. Weight and length of the children were measured with standard electronic scales and length boards. All measurement instruments were well maintained. We used the World Health Organization (WHO) growth standards to calculate *z* scores for measured body weight, length, head circumferences, and BMI at ages 6, 12, 24, and 36 months^32^. In addition to comparing *z* scores between the 2 groups, we also assessed children’s growth status based on the WHO’s percentile curve. Based on CDC’s recommendations, growth patterns that deviated for 2 SD (*z* score < −2, which corresponds to 2.3^rd^ percentile, or *z* score ≥ + 2, which corresponds to 97.9^th^ percentile) are considered nonstandard and may be associated with adverse health conditions. As an economic advantaged country, we do not have many children with failure to thrive or malnutrition^33^. Thus, association between breastfeeding and stunted growth was not further investigated in our study.

### Statistical methods

Comparisons of anthropometric measurements and laboratory results such as hemoglobin, ferritin, zinc, and vitamin D level between children of the eBF and MF groups were performed at ages 1, 2, and 3 years by student *t* or Fisher’s exact test. Binary logistic regression analyses were used to evaluate the association between exclusive breastfeeding and primary outcomes such as iron deficiency anemia and vitamin D insufficiency at age 1 year. Factors such as time of solid food introduction, birth body weight and height, gender, and maternal education were included in the regression analysis to compensate for potential confounders’ effects. All statistical analysis was carried out using the IBM SPSS Statistics Version 20 (Armonk, NY).

## Supplementary information

Supplement 1

Supplement 2
