# Supplementary material for: Trajectory of vitamin D, micronutrient status and childhood growth in exclusively breastfed children
Source: Sci Rep. 2019 Dec 13;9:19070. doi: 10.1038/s41598-019-55341-1 (PMC6910939; doi:10.1038/s41598-019-55341-1)
Supplement: Supplementary file 1 — Supplement 1 [file 41598_2019_55341_MOESM1_ESM.pdf]

Trajectory of vitamin D, micronutrient status and childhood growth in exclusively breastfed children

Sui-Ling Liao[1,2,5], MD, Tsung Chieh Yao[1,3,5], MD, PhD, Man-Chin Hua[1,2,5], MD, Ming-Han Tsai[1,2.5], MD, PhD, Shih-Yun Hsu[1,2], MD, Li-Chen Chen[1,3,5], MD, Kuo-Wei Yeh [1,3,5], MD, Chih-Yung Chiu[1,4,5], MD, PhD, Shen-Hao Lai[1,4,5*],MD, and Jing-Long Huang[1,3,5]

Supplement 1 Comparing participants who are lost to follow-up with those who continue to participate after ages 3 years

Characteristics	Participants (%)	Loss to f/u (%)	p
Sex (male)	238 (66)	160 (60)	0.12
Gestational Age (wk)	38.7 ± 1	38.5± 1	0.02
Birth body weight (g)	3180 ± 400	3140 ± 390	0.17
Birth body height (cm)	50.6 ± 2	50. 5± 2	0.64
Head circumference (cm)	33.8 ± 1.3	33.8 ± 1.4	0.87
Mode of delivery (NSD)	240 (66)	159 (59)	0.07
Mean BF duration (mo)	5.8 ± 8	5.4 ± 7	0.62
Time of solid food (mo)	5.1 ± 1	5.2 ± 1	0.22
Maternal body height (cm)	160 ± 6	159 ± 5	0.93
Maternal body weight (kg)*	59.6 ± 11	58.9 ± 10	0.79
Maternal Education			0.07
Secondary or high school	90 (25)	84 (31)	
College or above	272 (75)	184 (69)	

BF: breastfeeding.
NSD: natural spontaneous delivery
mo: months wk: weeks
f/u: follow-up

Total number of 3-year-old children with complete data: 362
Total number of children without complete data or lost to follow up after 3 years: 268
