# Supplementary material for: Trajectory of vitamin D, micronutrient status and childhood growth in exclusively breastfed children
Source: Sci Rep. 2019 Dec 13;9:19070. doi: 10.1038/s41598-019-55341-1 (PMC6910939; doi:10.1038/s41598-019-55341-1)
Supplement: Supplementary file 2 — Supplement 2 [file 41598_2019_55341_MOESM2_ESM.pdf]

Trajectory of vitamin D, micronutrient status and childhood growth in exclusively breastfed children

Sui-Ling Liao[1,2,5], MD, Tsung Chieh Yao[1,3,5], MD, PhD, Man-Chin Hua[1,2,5], MD, Ming-Han Tsai[1,2.5], MD, PhD, Shih-Yun Hsu[1,2], MD, Li-Chen Chen[1,3,5], MD, Kuo-Wei Yeh [1,3,5], MD, Chih-Yung Chiu[1,4,5], MD, PhD, Shen-Hao Lai[1,4,5*],MD, and Jing-Long Huang[1,3,5]

Supplement 2: Comparing solid food status between children of the different feeding groups

	MF n (%)	eBF n (%)	P
Time of solid food (mo)	5.1 ± 1	5.2 ± 1	0.20
Age 6 months			
Meat	41 (9.3)	21 (11.0)	0.52
Fish	61 (13.9.1)	27 (14.1)	0.94
Yolk	69 (15.8)	30 (15.7)	0.99
Age 12 months			
Meat	334 (83.9)	157 (84.9)	0.81
Fish	366 (92.0)	167 (90.3)	0.47
Yolk	318 (79.9)	156 (84.3)	0.21
Insufficient solid food	96 (24.1)	53 (28.6)	0.46

Rough estimation of solid food intake status after the age of 1 year was inquired and denoted as **insufficient solid food intake** if :

1. solid food was given for less than 2 meals per day and/or if amount per meal did not reach more than half bowl per meal (bowl ≒ 250g)
2. Children's main food source rely more on milk (either breastmilk or formula) than solid food.
